# Mitochondrial Quality Control: Role in Cardiac Models of Lethal Ischemia-Reperfusion Injury

**DOI:** 10.3390/cells9010214

**Published:** 2020-01-15

**Authors:** Andrew R. Kulek, Anthony Anzell, Joseph M. Wider, Thomas H. Sanderson, Karin Przyklenk

**Affiliations:** 1 Cardiovascular Research Institute, Wayne State University School of Medicine, Detroit, MI 48201, USA; at2028@wayne.edu (A.R.K.); aanzell@med.wayne.edu (A.A.); thsand@med.umich.edu (T.H.S.); 2 Department of Biochemistry, Microbiology and Immunology, Wayne State University School of Medicine, Detroit, MI 48201, USA; 3 Department of Physiology, Wayne State University School of Medicine, Detroit, MI 48201, USA; 4 Departments of Emergency Medicine and Molecular & Integrative Physiology, University of Michigan Medical School, Ann Arbor, MI 48109, USA; jwider@med.umich.edu; 5 Department of Emergency Medicine, Wayne State University School of Medicine, Detroit, MI 48201, USA

**Keywords:** heart, ischemia, reperfusion, mitochondria, fission, fusion, mitophagy

## Abstract

The current standard of care for acute myocardial infarction or ‘heart attack’ is timely restoration of blood flow to the ischemic region of the heart. While reperfusion is essential for the salvage of ischemic myocardium, re-introduction of blood flow paradoxically *kills* (rather than rescues) a population of previously ischemic cardiomyocytes—a phenomenon referred to as ‘lethal myocardial ischemia-reperfusion (IR) injury’. There is long-standing and exhaustive evidence that mitochondria are at the nexus of lethal IR injury. However, during the past decade, the paradigm of mitochondria as mediators of IR-induced cardiomyocyte death has been expanded to include the highly orchestrated process of mitochondrial quality control. Our aims in this review are to: (1) briefly summarize the current understanding of the pathogenesis of IR injury, and (2) incorporating landmark data from a broad spectrum of models (including immortalized cells, primary cardiomyocytes and intact hearts), provide a critical discussion of the emerging concept that mitochondrial dynamics and mitophagy (the components of mitochondrial quality control) may contribute to the pathogenesis of cardiomyocyte death in the setting of ischemia-reperfusion.

## 1. Introduction

Cardiovascular disease (CVD), comprising coronary heart disease (CHD), heart failure, stroke, and hypertension, is the leading global cause of death and disability, with roughly 18 million deaths attributed to CVD annually [1]. ‘Heart attack’, also referred to as acute myocardial infarction (MI), is a common and, in many instances, devastating outcome in patients with cardiovascular disease. The current gold standard for treating MI is rapid restoration of blood flow (reperfusion) to the ‘at risk’ myocardial tissue through primary percutaneous coronary intervention (PPCI) in combination with advanced anticoagulant and antiplatelet therapy [2,3,4,5,6]. The increased efficacy of this combination therapy paired with improved control of patient risk factors, has produced significant reductions in acute mortality rates from MI and coronary heart disease [7,8]. Despite this progress, MI continues to be a significant medical burden, as the prevalence of post-MI heart failure and long-term deleterious cardiac sequelae continue to rise [7,8].

**Figure 1:**
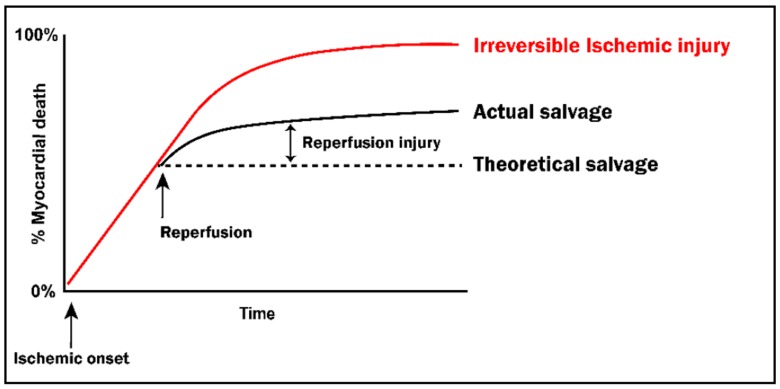
Myocardial ischemia-reperfusion injury. Schematic representation of myocardial cell death as a function of increasing duration of ischemia. In the absence of reperfusion, all cardiomyocytes will die: i.e., ~100% of cells are irreversibly injured (red curve). In theory, timely reintroduction of blood flow would salvage all remaining, previously ischemic cardiomyocytes (dotted line). However, reintroduction of blood flow paradoxically kills (rather than rescues) a population of previously ischemic myocytes—the phenomenon of ‘*lethal ischemia-reperfusion injury*’ (black curve). Adapted from reference [14].

MI is the result of an occlusion or blockage of one or more coronary vessels supplying a region of the heart, thereby depriving the myocardium of oxygen and nutrients and rendering the tissue distal to the site of occlusion. A hallmark of MI is the development of tissue necrosis (irreversible injury) by mechanisms involving ATP failure [9,10,11]. The myocardium is highly dependent on aerobic metabolism to generate sufficient amounts of ATP for maintenance of both cell viability and contractile function and, thus, is particularly sensitive to ischemic injury. This dependency on mitochondrial metabolic processes is reflected in the significant mitochondrial content of myocardial tissue, which accounts for >30% of the total volume of cardiomyocytes [12]. To mitigate the extent of necrosis that occurs during ischemia and promote myocardial tissue salvage, timely reperfusion of the ischemic tissue is absolutely necessary [3,6,7,13]. Indeed, restoration of blood flow is the current standard of care for the clinical treatment of acute MI. However, the re-instatement of blood flow to the ischemic tissue, while necessary for salvage, paradoxically kills (rather than rescues) a population of the previously ischemic myocytes—a phenomenon referred to as *lethal* ischemia-reperfusion (IR) injury [9,10,11] (Figure 1).

Substantial progress has been made in identifying the cellular events occurring upon relief of ischemia that may contribute to lethal IR injury, with mitochondrial integrity being central to this work [9,10,15,16]. Multiple mitochondria-centric mechanisms have been proposed to play a role, including (but not limited to): generation of reactive oxygen species (ROS), opening of the mitochondrial permeability transition pore (mPTP), and activation of intrinsic apoptosis [17,18,19,20,21,22]. More recently, the paradigm of mitochondria as mediators of lethal IR injury has been expanded to include the phenomenon of mitochondrial dynamics/morphosis, [23,24,25] and the concept that events leading to unbalanced mitochondrial fission-fusion are critical to the development of IR-induced cardiomyocyte death [25,26,27,28]. Our goals in this review are to: (1) briefly summarize the current understanding of the pathogenesis of lethal IR injury and, more specifically (2) focus on current and emerging evidence regarding the contribution of mitochondrial quality control (including mitochondrial morphosis and mitophagy) to ischemia-reperfusion-induced cardiomyocyte death.

## 2. Lethal Ischemia-Reperfusion Injury

### 2.1. The Trigger: Myocardial Ischemia

Evidence obtained from preclinical models and from clinical studies has demonstrated that, not surprisingly, the extent of cardiomyocyte death caused by myocardial ischemia *per se* is determined by both the magnitude of the deficit in oxygen supply and the duration of the ischemic insult [15]. After the onset of ischemia, there are two populations of cardiomyocytes in the ischemic territory: (1) *irreversibly* injured myoyctes that have undergone necrosis, and (2) *reversibly* injured myocytes that remain viable and have the potential to be salvaged upon reperfusion [9,29]. These two distinct injury populations are the result of spatial heterogeneity in both the sensitivity to ischemia and the severity of the ischemic insult (arising, for example, from varying degrees of collateral flow from adjacent coronary vessels) [9]. Moreover, and as expected, the proportion of irreversibly versus reversibly injured myocardium displays temporal variation and increases as the duration of ischemia is prolonged (Figure 1).

**Figure 2:**
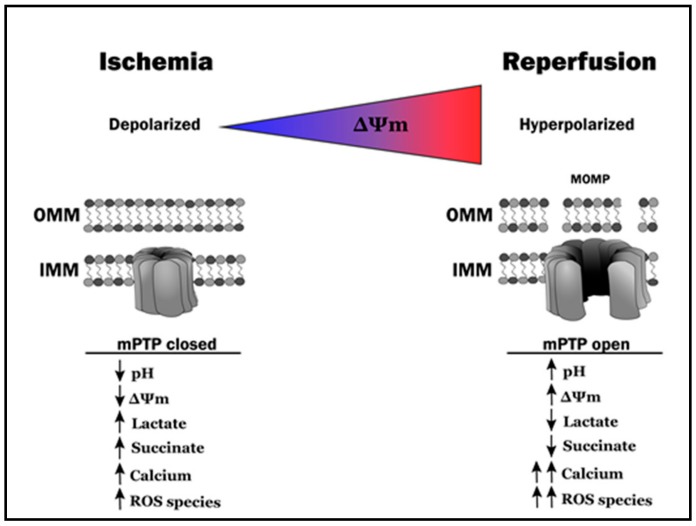
Cellular consequences of ischemia-reperfusion in cardiomyocytes. Under conditions of ischemia (left), mitochondria are depolarized (i.e., ΔΨm is decreased) and ATP stores are depleted. This is accompanied by acidosis secondary to lactate accumulation, and an increase in intracellular calcium concentration. However, the OMM remains intact and the mPTP remains closed. Reintroduction of oxygen (right), results in the raid normalization of pH and increase in ΔΨm, and precipitates multiple deleterious sequelae including generation of ROS, exacerbated calcium overload, disruption of the OMM and opening of the mPTP. OMM = outer mitochondrial membrane; IMM = inner mitochondrial membrane; MOMP = mitochondrial outer membrane permeabilization; mPTP = mitochondrial permeability transition pore; ΔΨm = mitochondrial membrane potential; ROS = reactive oxygen species. Adapted from reference [12].

The transition from reversible to irreversible injury during ischemia is the consequence of cellular events initiated by the ischemia-induced mismatch between myocardial oxygen supply and demand. These deleterious sequelae include (but are not limited to) the resultant shift from aerobic metabolism to anaerobic glycolysis, and subsequent inability to generate sufficient ATP to maintain ionic homeostasis and integrity of mitochondrial and sarcolemmal membranes [9,10,11,12,30,31,32,33,34,35,36,37,38,39,40,41] (Figure 2).

### 2.2. Reintroduction of Oxygen: A ‘Double-Edged Sword’

As discussed previously, reperfusion of the ischemic myocardium and the attendant reintroduction of oxygen and nutrients is required to salvage reversibly injured myocardium and limit infarct progression. However, for sub-populations of reversibly injured cardiomyocytes, re-instatement of blood flow paradoxically precipitates (rather than prevents) necrotic and apoptotic cell death [11,14,42]. The mechanisms of lethal IR injury are complex and multi-factorial and, despite decades of investigation, remain incompletely resolved [12,31,43,44,45]. There are, however, two recurring themes. First, mitochondria, and loss of mitochondrial integrity, have been identified to play a pivotal role, with emphasis to date focusing largely on the well-documented cytotoxic consequences of mitochondrial ROS production and opening of the mPTP at the time of reoxygenation [12,16,43,46,47,48,49] (Figure 2). Second, despite the wealth of evidence obtained in preclinical models for the contribution of these mitochondria-centric mechanisms to the pathogenesis of lethal IR injury, efforts to translate these insights into clinical therapies for the treatment of acute MI have been unsuccessful: i.e., pharmacologic therapies aimed at scavenging ROS and preventing mPTP opening at the time of reperfusion have failed to improve outcomes [50,51,52,53,54]. These data underscore the importance of expanding our understanding of the molecular mechanisms of lethal IR injury, with the goal of identifying novel and rationally designed pharmacologic approaches to attenuate the deleterious component of reperfusion. In this regard, increasing attention has focused on the possible role of mitochondrial morphosis and mitophagy in IR-induced cell death, and manipulation of the key protein mediators of inner and outer mitochondrial membrane integrity as targets for intervention.

## 3. Mitochondrial Morphosis

### 3.1. Definitions and Key Players

A wealth of evidence over the past two decades has demonstrated that mitochondria are not discrete and static organelles. Rather, mitochondria are highly dynamic, undergoing adaptive changes in shape and ultrastructure (i.e., fission [division] and fusion) in response to cellular stress and resultant alterations in intracellular environment—a process that is collectively termed ‘mitochondrial morphosis’ [55]. Cell cycle progression and cellular differentiation, oxidative stress, metabolic perturbations and induction of programmed death pathways are all characterized by transient states of fission and fusion [56,57]. Mitochondria are not synthesized *de novo*; rather, fission-fusion—followed by the process of mitophagy discussed in later sections of this review—are critical in ensuring that damaged and dysfunctional mitochondria are culled from cells and that mutated mtDNA is not propagated [58,59,60,61,62,63].

Initial studies conducted in yeast revealed that mitochondrial morphology and metabolic state are highly integrated, with fused networks of mitochondria displaying more efficient respiratory capacity and increased ATP generation [64,65]. This concept has subsequently been confirmed in mammalian cells; indeed, in tissues with a high metabolic demand such as heart, tight control of mitochondrial function and integrity is particularly critical. The molecular machinery required for fission and fusion has been confirmed to be present in immature neonatal cardiomyocytes, in cardiac cell lines (including HL-1 and H9c2 cells) and, importantly, in mature cardiomyocytes where spatial constraints are imposed by the architecture of the myofilaments and T-tubules [57]. Specifically, in the adult heart, mitochondria are confined to the interfibrillar, subsarcolemmal, and perinuclear regions. These compartmentalized mitochondrial populations are strategically oriented so that calcium homeostasis and ATP generation are spatially linked to the contractile machinery, the sarcoplasmic reticulum, and T-tubules [62]. Consequences of mitochondrial dysfunction in the heart include dysregulation of calcium homeostasis, endoplasmic reticulum (ER) stress and impaired contractility [9,15,66,67,68,69]. Moreover, there is growing evidence that dysregulation of fission-fusion may contribute to the maladaptive changes underlying heart failure, cardiomyopathies, cardiac hypertrophy and adverse remodeling—and, as discussed in this review, cardiomyocyte fate in the setting of acute myocardial infarction [70,71,72,73].

### 3.2. Mitochondrial Fission: Sacrificing One for the Team

**Figure 3:**
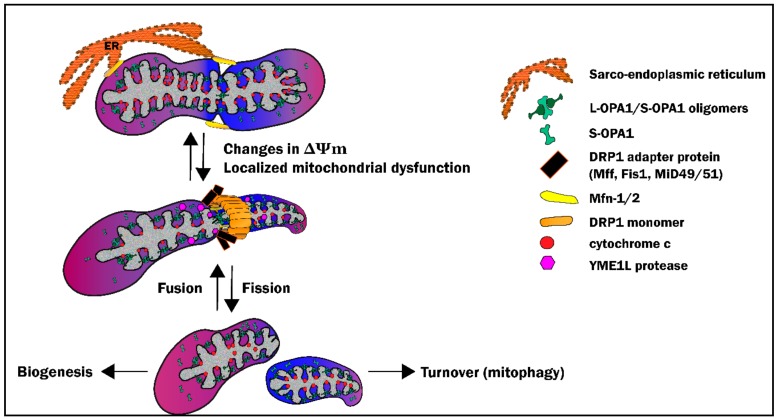
Mitochondrial morphosis under physiologic conditions. The dynamic fission-fusion balance occurs under steady-state conditions to promote mitochondrial biogenesis and/or culling of dysfunctional mitochondria by mitophagy. DRP1-mediated fission at the outer mitochondrial membrane reportedly occurs through interaction with cognate adapter proteins (Mff, Fis1, MiD49/51) at specific ER-contact foci. Transient cycling of DRP1 to these foci is suggested to maintain the fission-fusion balance. Dysfunctional mitochondrial segments are represented in blue, viable mitochondria represented in purple. OPA1 = optic atrophy protein-1; DRP1 = Dynamin related protein-1; Mfn 1/2 = mitofusins 1 & 2.

Mechanistic insight into mitochondrial morphosis was initially obtained in yeast and, more recently, in neuronal cells [74,75,76,77,78,79,80,81,82,83,84,85,86,87], with genetic manipulation of yeast homologues of the mammalian guanosine triphosphates (GTPases) providing the foundation for our current understanding of the molecular mediators of fission and fusion [63,65,74,88]. In a process that is highly conserved across species, mitochondrial fission, when balanced with fusion, is a normal physiologic process that: (1) serves as the first step in the culling of damaged and dysfunctional organelles from the mitochondrial network, and (2) fragments the network to facilitate trafficking of mitochondria to microdomains within the cell [62,87,89,90,91,92,93,94] (Figure 3). Excessive fission, is however, pathologic: i.e., is associated with release of cytochrome *c* into the cytosol and initiation of apoptosis [25,63,95,96].

Fission events are localized at the outer mitochondrial membrane (OMM) and are under the control of Dynamin related protein-1 (DRP1), the “master regulator” of mitochondrial fission [79,94,97] (Figure 3). The DRP1 protein consists of three domains: (1) a GTPase domain, (2) a central domain, and (3) a GTPase effector domain (GED). Under homeostatic conditions, DRP1 is primarily distributed in the cytoplasm. However, in response to physiologic or pathophysiologic stimuli and resultant changes in cellular ATP and calcium concentrations, DRP1 is triggered to translocate to the OMM through protein kinase A- and calcineurin-mediated post-translational modifications primarily involving phosphorylation/dephosphorylation at serine residues (Ser 616 and Ser637) within the GED domain [56,94,98,99,100,101]. DRP1 moieties then oligomerize to form a helical “fission complex”, or ring-like multimeric structure encircling the mitochondria OMM. To complete the fission event, the GTPase domain of DRP1 utilizes GTP hydrolysis to constrict the OMM at specific contact points, pinching off mitochondria [98,102,103,104] (Figure 3).

DRP1 does not contain either a mitochondrial targeting sequence (MTS) or membrane-localizing pleckstrin homology domain. Accordingly, OMM-anchored adaptor proteins, including human fission protein (hFis1), mitochondrial fission factor (Mff), and mitochondrial-dynamics proteins (MiD49/MiD51), mediate DRP1 recruitment [75,105,106,107,108] (Figure 3). In mammalian cells, the specific role of each of these adaptor proteins is poorly understood [107,108,109,110], with current evidence suggesting that hFis1 is responsible for DRP1 recruitment during pathological fission whereas Mff and MiD49/51 assume a predominant role during physiological fission [24,75,107,108,111,112,113]. DRP1-mediated fission events may therefore require a complex interplay of these adapter proteins to facilitate DRP1 docking to the OMM and mediate assembly of the fission complex.

### 3.3. Mitochondrial Outer Membrane Permeabilization: When Fission Leads to Death

**Figure 4:**
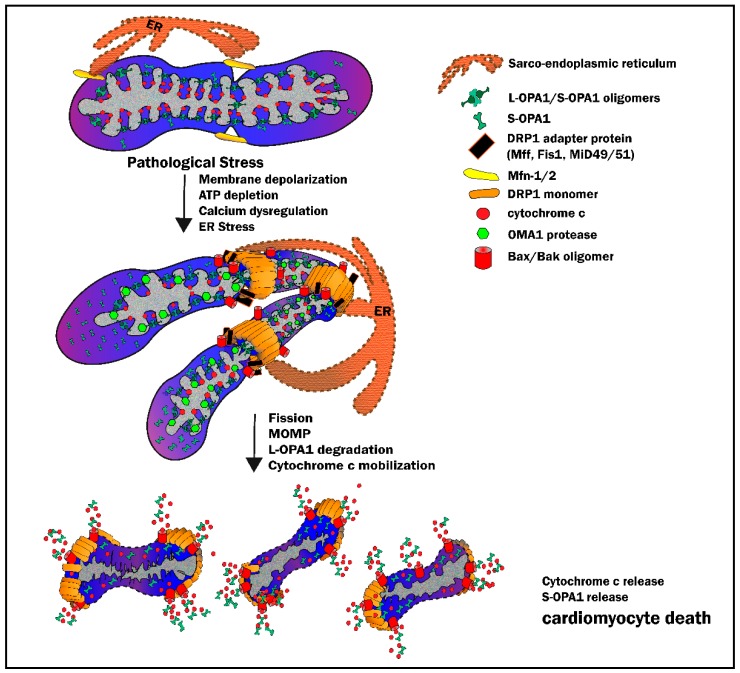
Mitochondrial morphosis under pathologic conditions. Ischemia-reperfusion is associated with an increase in DRP1-mediated fission, mitochondrial outer membrane permeabilization (MOMP) and the release of apoptogenic species into the cytoplasm. Degradation of OPA1 oligomers (achieved via stress-associated OMA1-mediated proteolytic cleavage of OPA1) disrupts cristae architecture, dissociating cytochrome *c* from within the cristae folds. MOMP is proposed to be a consequence of interaction of DRP1 with Bax/Bak oligomers at the outer membrane, and is considered to be a lethal event. Dysfunctional mitochondrial segments are represented in blue, viable mitochondria represented in purple. OPA1 = optic atrophy protein-1; DRP1 = dynamin related protein-1; Mfn 1/2 = mitofusins 1 & 2; MOMP = mitochondrial outer membrane permeabilization.

The intrinsic, mitochondrial-initiated apoptosis pathway is characterized by excessive fission and increased permeability of mitochondrial membranes [114], with DRP1 being well-recognized to have a focal role in mediating the process of mitochondrial outer membrane permeabilization (MOMP) [115,116,117] (Figure 4). This loss of mitochondrial outer membrane integrity is widely considered to be the “point of no return” in intrinsic apoptosis, with the Bcl-2 family proteins (including Bax, Bak and tBID) being pivotal in this process [118]. In brief: DRP1 translocation to the mitochondria promotes Bax/Bak recruitment, oligomerization and pore formation at the OMM, thereby triggering the release of cytochrome *c* [117,119]. Moreover, Bax/Bak recruitment to the OMM is accompanied by the release of a novel apoptogenic factor (DDP/TIMM8a) that facilitates an amplified DRP1 recruitment to Bax/Bak sites where DRP1 is then stabilized by Bax/Bak-dependent sumoylation [120,121] (Figure 4). The critical role of DRP1 in this process is illustrated by observations that, in the settings of DRP1 inhibition achieved by knockdown or overexpression of dominant negative mutant forms of the protein, DRP1 docking to the OMM is impaired and release of cytochrome *c* is prevented or delayed [114,122,123]. Finally, DRP1-mediated fission, and the interaction between DRP1 and Bax/Bak oligomers, also involves mitochondrial-ER contact sites along the OMM [66,69,124,125] (Figure 4), with ER stress and ER-mediated calcium release being sufficient to serve as stimuli for DRP1 recruitment and initiation of Bax/Bak oligomerization and MOMP [66,125]. Taken together, these observations underscore the concept that DRP1 (and DRP recruitment to mitochondria) is at the nexus of mitochondrial quality control, pathologic fission, and apoptotic cell death via the intrinsic pathway.

### 3.4. Mitochondrial Fusion: Safety in Numbers

Mitochondrial fusion and the formation of mitochondrial networks is associated with efficient respiration and ATP production [126,127,128,129,130,131]. The fusion process requires dedicated machinery at both the inner and outer membranes, and includes three evolutionarily conserved GTPases: mitofusin 1 (Mfn1), mitofusin 2 (Mfn2) and optic atrophy protein (OPA1) [132,133,134,135,136,137]. Mfn1 and Mfn2 share > 75% homology, and both are OMM transmembrane proteins responsible for OMM fusion: i.e., the tethering of adjacent mitochondrial outer membranes via GTP hydrolysis. Interestingly, Mfn2 has been implicated to have a second role, serving as an intimate docking site between mitochondria and the ER that facilitates efficient mitochondrial calcium buffering [132,134,136,138,139,140,141]. The third protein, OPA1, is a member of the Dynamin superfamily, is located at the inner mitochondrial membrane (IMM), and is the regulator of IMM fusion [62,142,143]. In addition—and of particular relevance to the overarching topic of mitochondrial quality control—OPA1 is also critical for: (1) cristae morphogenesis and the bridging of adjacent cristae folds, (2) maintenance of cristae architecture, (3) regulation of respiratory supercomplex assembly and (4) tethering of cytochrome *c* within the mitochondrial cristae [126,129,130,135,143,144,145]. While OPA1 acts in concert with Mfn1 (but not Mfn2) to achieve fusion of the IMM, maintenance of cristae architecture is strictly reliant on OPA1 GTPase activity [135,144,146].

OPA1 function is tightly regulated at both the transcriptional level (through alternative splicing of three of the OPA1 exons) and post-transcriptionally via proteolytic cleavage at two distinct peptide sequences (S1 and S2) [146,147]. The mammalian OPA1 gene generates eight mRNA splice variants that all retain the catalytic GTPase domain. Splice variants are then subject to proteolytic processing in order to generate cell-specific profiles of OPA1 peptides (i.e., OPA1 forms). This hierarchical regulation for OPA1 at the RNA and protein level gives multiple layers of control over the IMM and cristae morphology [147,148,149,150].

**Figure 5:**
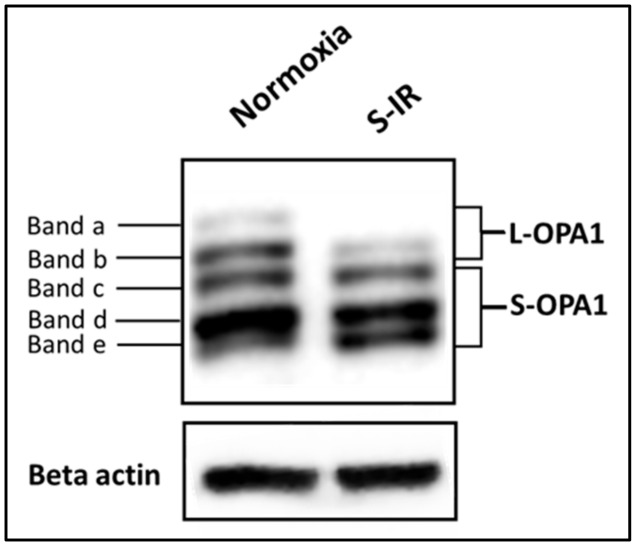
Example of OPA1 expression in whole cell lysates obtained from HL-1 cardiomyocytes under normoxic conditions and following simulated ischemia-reperfusion. HL-1 cardiomyocytes display five distinct OPA1 forms (bands a–e: 75–100 kDa), including two long (L) forms (bands a,b) and three short (S) forms (bands c–e). Ischemia-reperfusion is associated with the reduced expression of L-OPA1 bands due to OMA1-mediated cleavage of OPA1 and an attendant increase in expression of S-OPA1. S-IR = simulated ischemia-reperfusion.

Cardiomyocytes constitutively express five OPA1 forms in the range of 75-100 kDa and detected by immunoblotting as five distinct bands (denoted as bands a–e) [56,72,148,151,152] (Figure 5). These five molecular weight bands are grouped into higher molecular weight long OPA1 (L-OPA1, bands a/b, ~100 kDa) and lower molecular weight short OPA1 (S-OPA1, bands c,d,e, ~75–85 kDa) (Figure 5). Both L-OPA1 bands contain an S1 cleavage site, capable of generating S-OPA1 bands c and e, whereas a single L-OPA1 band contains an S2 cleavage site, capable of generating S-OPA1, band-d [56,147,153].

The fact that the L-OPA1 forms contain two distinct cleavage sites (S1 and S2) is important, as this gives constitutive and inducible control over OPA1 processing. The proteases implicated in mammalian OPA1 form generation are YME1L (a constitutively active, ATP-dependent protease in the intermembrane space) and OMA1 (a stress-activated zinc metalloproteinase residing in the matrix). Each protease recognizes a cognate cleavage sequence within OPA1: S1 (for OMA1 cleavage) and S2 (for YME1L cleavage) [131,146,147,153,154,155]. Constitutive cleavage through the activity of YME1L at the L-OPA1 S2 site is necessary to generate S-OPA1 peptides that facilitate oligomerization with L-OPA1 and bridge adjacent cristae, a process that is required for the tight regulation of cristae architecture in response to changes in oxidative phosphorylation and ATP demand, thereby linking metabolism to mitochondrial structure [131,147,156]. In contrast, inducible OMA1 activity is the result of pathological dissipation of the mitochondrial membrane potential or injury and activation of apoptosis [146,147,149]. However, despite these distinctions between the two proteases, data obtained with genetic deletion of YME1L and OMA1, individually or in combination, has revealed evidence of reciprocity between the two enzymes: i.e., cleavage of OPA1 by OMA1 is not limited exclusively to conditions of stress, but also reportedly occurs in the setting of YME1L inhibition [147,157] (see additional details below).

### 3.5. Disruption of Cristae Architecture: Opening the Cytochrome C Flood Gates

During induction of apoptosis, there are disruptions in IMM architecture, including inducible OMA1-mediated cleavage of OPA1, that facilitate the release of cytochrome *c* and other apoptogenic factors into the cytosol. This is supported by experiments in which genetic depletion of OPA1 is associated with cristae disorganization and predisposes the cells to apoptosis, while, conversely ectopic overexpression of OPA1 confers resistance to apoptotic cell death [146,158]. Moreover, and perhaps not surprisingly, stress-induced OPA1 cleavage promotes cytochrome *c* mobilization and apoptosis [144,145,148,157,159,160].

Despite the compelling evidence that L-OPA1 oligomerization with S-OPA1 is necessary for maintaining cristae architecture [144,146], there are data to suggest that L-OPA1 alone, or the presence of the S-OPA1 band d alone, are sufficient for inner membrane fusion [72,147,148]. Investigation of the reciprocal regulation of YME1L and OMA1 has demonstrated that the type of stress dictates the stability of these two proteases and, thus, OPA1 profile. When ATP is available in the presence of cellular stress (i.e., mitochondrial depolarization), YME1L is active and degrades OMA1, while the combined stress of membrane depolarization together with ATP depletion favors OMA1 stabilization [147,153]. This is significant in the context of apoptosis, as S-OPA1 bands c/e generated by OMA1 proteolysis are purportedly pro-fission and promote aberrant cristae remodeling [147,159]. Furthermore, it has been suggested that release of specific S-OPA1 fragments from stressed mitochondria into the cytosol is required for the rapid and complete release of cytochrome *c* [149,161,162].

Two critical events in the apoptotic cascade, MOMP and opening of the mPTP, are intimately connected to the maintenance of cristae architecture by OPA1. For example, Bax/Bak-mediated MOMP has been shown to occur in response to transmission of ER stress to the mitochondria and subsequent sequestration of calcium within the mitochondrial matrix. These hallmarks of apoptosis (i.e., matrix swelling and calcium overload) are, in turn, accompanied by disruption of cristae architecture, loss of mitochondrial membrane potential and OPA1 degradation [66,124,163]. Moreover, putative triggers of MOMP, including Bax/Bak and DRP1, have been shown to regulate cristae morphology by induction of OMA1 and destabilization of OPA1 oligomers—a sequence of events that mobilize cytochrome *c* release from cristae [156,159]. Given that a reported 80% of cytochrome *c* is bound within the cristae by OPA1, apoptosis is highly dependent on IMM architecture and OPA1 integrity [144,160,161].

### 3.6. A Complex Web: Ischemia-Reperfusion, Metabolic Dysfunction and Mitochondrial Morphosis

As noted in Section 2 and Section 3, ischemia has profound and well-documented effects on myocardial metabolism. In brief: the diminished delivery of oxygen and nutrients (including glucose and fatty acids) to cardiomyocytes results in a metabolic shift from aerobic oxidative phosphorylation to anaerobic glycolysis and attendant deficit in ATP production (see Section 2.1 and Figure 2). The resultant depletion of high energy phosphate stores is accompanied by an inability to maintain mitochondrial membrane potential and ionic homeostasis (Figure 2). Most notably, activity of myocardial ATPase enzymes (including, in particular, the sarcoplasmic reticulum Ca^2+^ ATPase (SERCA) and the Na^+^/K^+^ ATPase) are impaired, culminating in the dysregulation of calcium homeostasis via accumulation of intracellular calcium through the Na^+^/Ca^2+^ exchanger (a condition that is exacerbated in the setting of acidosis and lactate accumulation), mitochondrial calcium sequestration from the cytosol (in the face of dysfunctional SERCA) and sarco-endoplasmic reticular calcium transmission to the mitochondria [33,35,36,37,164,165].

Failure to maintain calcium homeostasis—a consequence of the aforementioned metabolic derangements, and the hallmark of lethal ischemia-reperfusion injury—also plays a role in regulating mitochondrial quality control. Calcium overload activates proteases and phosphatases that modify components of the electron transport chain as well as key molecular GTPases involved in mitochondrial morphosis [99,165,166,167,168], effectively priming the ischemic myocardium for the hyperpolarization of mitochondrial membranes, burst of ROS production, disruption of the outer mitochondrial membrane and opening of the mPTP that occurs upon reperfusion and reintroduction of oxygen (Figure 2) [164,169,170]. These metabolic sequalae orchestrate inner and outer mitochondrial membrane reorganization, largely through activation of the calcium-dependent phosphatase, calcineurin [99,101,171], and the inner mitochondrial membrane protease, OMA1 [151,153,157,172]. In addition, calcineurin activation results in the dephosphorylation of DRP1, thereby favoring fission [26,28,99], while OMA1 activation results in the proteolytic cleavage of OPA1 and resultant disruption of cristae architecture [151,153,157,172]. Finally, collapse of mitochondrial membrane potential and compromised mitochondrial integrity have been identified as drivers for pathological fission [28,173]. Thus, and perhaps not surprisingly, metabolic perturbations and changes in mitochondrial phenotype are integrated (rather than discrete) consequences of myocardial ischemia-reperfusion.

## 4. Mitochondrial Dynamics and Cardiomyocyte Fate

As summarized in Section 2, there is long-standing evidence that mitochondria are at the epicenter of lethal myocardial ischemia-reperfusion injury. Historically, considerable attention focused on mitochondria as both a source and target of cytotoxic, reperfusion-induced ROS generation and, more recently, on the status of the mPTP [12,20,25,47,174,175,176]. However, over the past decade, the paradigm of ‘mitochondria as determinants of IR injury’ has expanded to include the concept that mitochondrial dynamics (or, more specifically, a pathologic imbalance between fission-fusion) may play a causal, mechanistic role in determining the fate of cardiomyocytes subjected to IR [25,26,28,71,72,177].

### 4.1. IR Injury and the Outer Mitochondrial Membrane—DRP1-Mediated Fission

It is well-established that, in cells exposed to a noxious stressor, DRP1 (and recruitment of DRP1 to the OMM) is intricately linked with the processes of intrinsic apoptosis and MOMP (see Section 3.3) [68,69,70,114,115,116,117,118,119,122,123,124,125,163,178]. The question is: does DRP1-mediated fission contribute to lethal IR-induced cardiomyocyte death?

Data obtained in multiple models has demonstrated that restoration of oxygen to ischemic cardiomyocytes is associated with the rapid (within <5 min) translocation of DRP1 from the cytosol to mitochondria [24,26,28,56,100,101], presumably in response to post-translational modification of the GED domain (see Section 3.2: [99,100,101,171,179,180,181]). Cellular redistribution of DRP1 was accompanied by release of cytochrome *c* from mitochondria into the cytosol, subsequent cleavage of caspase 3 (a harbinger of apoptosis) and, most notably, mitochondrial fragmentation [23,24,26,28,100,182,183] thereby implying an *association* between DRP1 translocation to the OMM, mitochondrial fission and cardiomyocyte death. If DRP1-mediated fission plays a *causal* role in cardiomyocyte death, one would expect that pharmacologic inhibition or genetic silencing of DRP1 would result in better maintenance of cardiomyocyte viability following ischemia-reperfusion. In apparent support of this concept, pre-ischemic administration of the agents MDIVI-1 (mitochondrial division inhibitor-1: the putative archetypal small molecule inhibitor of DRP1), P110 and Dynasore, as well as siRNA-mediated knockdown of DRP1 and transfection with a dominant negative DRP1 mutant, have all been show to attenuate mitochondrial fragmentation, improve cardiomyocyte viability in cell culture models of IR, and reduce infarct volumes in in vivo models of acute MI [23,24,26,28,123,184,185].

While these data are consistent with the concept of cause-and-effect, there are two important caveats to these observations. First, although MDIVI-1 has been regarded as a selective inhibitor of DRP1, recent studies revealed that MDIVI-1 does not directly inhibit DRP1 GTPase activity, nor does it attenuate mitochondrial fragmentation in cells challenged by exposure to staurosporine. Rather, MDIVI-1 inhibited reverse electron transport through mitochondrial Complex 1, a classic source of ROS production during early reperfusion [186]. Second, while MDIVI-1 administered before the onset of ischemia-reperfusion is cardioprotective, treatment initiated at the time of reperfusion *exacerbated* cardiomyocyte death in HL-1 cardiomyocytes subjected to IR [28] and failed to reduce infarct size in the translationally relevant, swine model of acute MI [187].

### 4.2. IR Injury and the Inner Mitochondrial Membrane—OPA1 and Cristae Integrity

The molecular consequences of myocardial ischemia-reperfusion are not limited to the aforementioned events occurring at the OMM. Rather, results obtained in multiple cell types (including cardiomyocytes) have demonstrated that IR initiates proteolysis of L-OPA1 forms, achieved via inducible cleavage by OMA1 and yielding S-OPA1 bands c/e [148,151,188]. The S-OPA1 forms then translocate from the intermembrane space into the cytosol, where they purportedly promote fission [161].

These data raise two questions: does disruption of OPA1 play a causal role in IR-induced cardiomyocyte death and, if so, which of the two distinct roles of OPA1 (maintenance of cristae architecture versus regulator of IMM fusion [129,135,143,144,145,146], is critical in determining cardiomyocyte fate? With regard to the first issue, there is general agreement among the still-limited number of studies conducted to date that lethal IR injury is exacerbated in models of OPA1 knockdown [71,72,128,152,189,190]. This is illustrated by data obtained using a novel mouse model displaying a ~70% reduction in OPA1 protein expression: infarct size following coronary artery occlusion-reperfusion in vivo, or following global ischemia-reperfusion ex vivo, was increased when compared with wild-type controls [189]. Interestingly, there were no differences in markers of apoptosis between the OPA knockdown and wild-type cohorts, suggesting that the exacerbated cell death was a consequence of necrosis or a non-canonical programmed cell death pathway (i.e., not apoptosis) [189]. Corroborating evidence has been obtained from additional studies that used the converse approach [128,145,158]). For example, release of lactate dehydrogenase (a surrogate index of cardiomyocyte death) following ex vivo ischemia-reperfusion was attenuated in hearts from transgenic *Opa1* mice characterized by a ~1.5-fold increase in OPA1 protein expression versus hearts from wild-type animals [145]. However, in apparent contrast, ~50% overexpression of OPA1 protein expression in H9c2 cells had the expected effect of attenuating the fragmentation of mitochondria in response to IR but failed to protect against IR-induced apoptosis [152]. The second question—the comparative importance of OPA1-mediated maintenance of IMM cristae architecture versus OPA1-mediated IMM fusion in the relationship between OPA1 expression and cardiomyocyte viability in the setting of lethal IR injury—has not been resolved. Insight into this issue may, however, be inferred from the evidence that OPA1 is responsible for tethering cytochrome *c* (the canonical trigger for apoptosis) to the IMM [145,152,189], thereby potentially favoring the role of cristae integrity in this paradigm.

### 4.3. Importance of Inner Versus Outer Mitochondrial Membrane Integrity in Lethal Ir Injury?

As summarized above, compelling data have been obtained to implicate events at both the OMM and IMM in IR-induced cardiomyocyte death. This raises an obvious and potentially important question for the targeted development of novel mitochondrial-centric therapies to attenuate lethal IR injury: is there a critical step—DRP1-mediated fission, the role of DRP1 in MOMP, or loss of cristae integrity due to OPA1 disruption—that serves as the lynchpin and *de facto* ‘executioner’ for cardiomyocytes subjected to ischemia-reperfusion?

Initial studies identified pathologic recruitment of DRP1 and resultant outer membrane fission as the default event precipitating cell death [24,26,28,123,184]. Indeed, from a temporal perspective, DRP1 translocation to the OMM precedes cytochrome *c* release and apoptosis following relief of ischemia [24,25,26,27,28,123,185]. There is, however, a complex interplay between DRP1 translocation, Bax/Bak recruitment and oligomerization at the OMM, and subsequent outer membrane permeabilization [66,115,116,117,118,121,122,191] (see Section 3.3). This is underscored by observations that, while Bax/Bak-mediated MOMP is purportedly necessary and sufficient for release of apoptogenic factors from mitochondria, genetic inhibition of DRP1 has been shown to prevent or delay the release of cytochrome *c* into the cytosol [28,115,116,163,180,192]. Resolution of this issue is further complicated by the fact that there is molecular interaction between the OMM and IMM. For example, DRP1 and Bax/Bak reportedly participate in remodeling IMM cristae architecture through mechanisms that at least in part, involve OMA1 [156,159,160,161]. Finally, for cytochrome *c* to be released into the cytosol, it must presumably dissociate from the cristae, thereby implying a defect or disruption in OPA1 and its ability to bind cytochrome *c* to the cristae [161]. Given these multifaceted interactions among the key molecular determinants of OMM and IMM integrity, resolution of the site and identity of a single ‘executioner’ may, at best, be challenging.

## 5. Pharmacologic Targeting of Mitochondrial Morphosis to Attenuate Lethal IR Injury

Despite the continued gaps in our understanding of the precise mechanisms(s) by which aberrant fission-fusion contributes to IR-induced cardiomyocyte death, the data raise the intriguing possibility that the development of pharmacologic agents capable of targeting DRP1-mediated fission, DRP1-associated MOMP, and/or loss of cristae integrity may potentially provide novel strategies to attenuate lethal ischemia-reperfusion injury and improve outcomes in patients post-MI.

The earliest studies aimed at pharmacologically modulating IR-induced fission focused on DRP1 and administration of the putative small molecule inhibitor, MDIVI-1. As discussed previously, pretreatment with MDIVI-1 was consistently reported to be cardioprotective [23,25,26,28,119], whereas treatment initiated at the time of reoxygenation was ineffective in attenuating lethal IR injury [28,187]. However, and contrary to the premise of the initial studies with this agent, MDIVI-1 is, in fact, not a direct and selective inhibitor of DRP1 GTPase activity [186]. Rather, the protective effects of MDIVI-1 are reportedly a consequence of inhibition of reverse electron transport through Complex 1 and, in turn, attenuation of ROS production during IR [186]. This latter observation does not undermine the favorable outcomes obtained with MDIVI-1 pretreatment, but is problematic in terms of potential clinical application as acute MI is an unanticipated event and prophylaxis is not feasible.

More recently, two additional and presumably more appropriately targeted inhibitors have been developed. The first, P110, is a selective peptide inhibitor of the interaction between DRP1 and its cognate mitochondrial receptor, Fis1. This agent has been evaluated in models of IR and cardiac arrest and shown to inhibit DRP1-mediated fission, diminish ROS generation and release of cytochrome *c*, attenuate indices of mitochondrial dysfunction and reduce myocardial infarct size [24,185]. The second candidate, Dynasore, is a small molecule, non-competitive DRP1 inhibitor, and similar cardioprotective effects have been described with administration of the agent *in both* in vitro and ex vivo models of lethal IR injury [184].

A final and novel pharmacologic approach has focused on inhibition of the stress-induced protease OMA1 in an attempt to better-maintain OPA1 integrity and preserve cristae architecture [188]. Administration of epigallocatechin gallate (EGCG), a polyphenol present in green tea, attenuated OPA1 cleavage and loss of cristae integrity and reduced cytochrome *c* release and indices of apoptosis in neonatal cardiomyocytes subjected to IR [188]. While these data are intriguing, no data are provided to establish that EGCG is selective for OMA1. In addition, robust in vivo evaluation of EGCG and confirmation of the purported benefits of the agent in translationally relevant models of lethal ischemia-reperfusion injury is clearly required.

## 6. Mitophagy

The second and subsequent component of mitochondrial quality control is mitophagy: i.e., the selective form of macroautophagy that is specifically responsible for the degradation of mitochondria. Of particular interest, both mitochondrial dynamics and mitophagy have been proposed to have a causal, mechanistic role in lethal ischemia-reperfusion injury.

### 6.1. Definitions and Key Players

**Figure 6:**
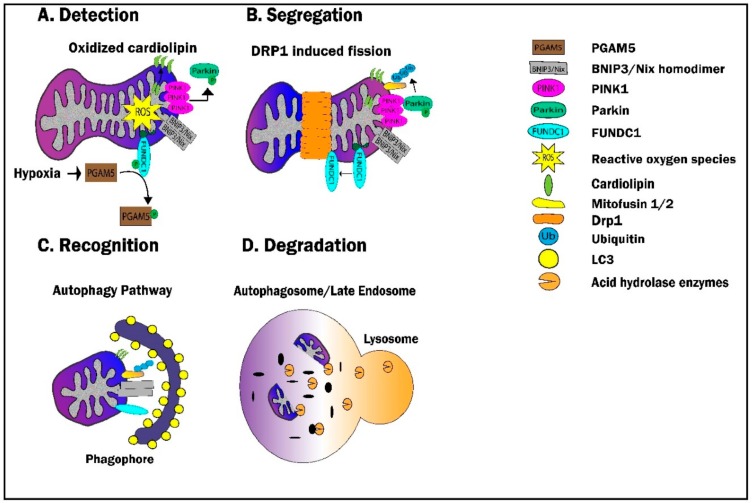
Mitophagic elimination of dysfunctional mitochondria during ischemia-reperfusion. Mitophagy is achieved via four distinct pathways: (**A**) *Detection* of dysfunctional mitochondria through PINK1/Parkin recruitment, BNIP3/Nix homodimerization/activation, FUNDC1 activation, and externalization of cardiolipin. (**B**) *Segregation* of dysfunctional mitochondria through DRP1-dependent fission and disruption of fusion machinery. (**C**) *Recognition* of the dysfunctional mitochondria via LC3-binding domain interactions with the phagophore. (**D**) Degradation of the sequestered mitochondria by acid hydrolase enzymes following lysosomal fusion. Dysfunctional mitochondrial segments are represented in blue, viable mitochondria represented in purple. PGAM5 = positive regulator of FUNDC1; PINK1 = PTEN-induced kinase; BNIP3 = Bcl-2 adenovirus E1B 19-kDa-interacting protein-3; Mfn 1/2 = Mitofusins 1 & 2; DRP1 = dynamin related protein-1; LC3 = light chain-3 protein; ROS = reactive oxygen species.

As discussed in detail in Section 3, mitochondrial dysfunction is well-recognized to be a precursor to cell death via the induction of apoptosis. Therefore, it is essential to maintain a healthy mitochondrial network by eliminating damaged mitochondria via mitophagy. Currently, four pathways have been identified to carry out mitophagy which include: (1) the PINK1/Parkin pathway, (2) the BNIP3/Nix pathway, (3) the FUNDC1 pathway, and (4) the Cardiolipin pathway. In concert with mitochondrial biogenesis, mitophagy ensures a healthy mitochondrial network through mitochondrial turnover. The pathways of mitophagy are complex and dynamic processes and have been the topic of recent, in-depth reviews [193,194,195,196,197]. However, and as described below, all pathways share the following four required steps: (1) the detection of dysfunctional mitochondria, (2) segregation of dysfunctional mitochondria from the healthy mitochondrial network, (3) recognition/sequestration of defective mitochondria by autophagosomes, and (4) degradation via lysosomal enzymes (Figure 6).

#### 6.1.1. Detection

Disruption of the IMM due to chronic hypoxia or acute IR injury is the major driver of mitophagy [198,199,200,201,202]. In the setting of IR, destabilization of the IMM (together with multiple other deleterious sequelae) is largely a consequence of ROS generation and subsequent oxidative damage to mitochondrial lipids and proteins [166,203]. IMM destabilization leads to the activation of the PINK1/Parkin pathway via the accumulation of PTEN-induced kinase (PINK1) on depolarized mitochondria [204,205]. In a highly orchestrated process, PINK1 then recruits and activates Parkin via phosphorylation [205,206,207,208,209,210,211,212], which, in its role as an E3 ubiquitin ligase, ubiquitinates outer mitochondrial membrane proteins [213,214,215] (Figure 6). This classic paradigm of PINK1/Parkin activation is not, however, the sole mechanism that serves to identify and target defective mitochondria. For example, the generation of ROS can also lead to the oxidation, redistribution, and externalization of the mitochondrial lipid, cardiolipin, which reportedly can act as a ‘mitophagy receptor’ [216,217,218]. In addition, Bcl-2 adenovirus E1B 19 kDa-interacting protein 3 (BNIP3) and its homologue Nix function as a sensor of mitochondrial oxidative stress in response to IR [69,182,218,219,220], with the oxidation of the N-terminal cysteine residue of BNIP3 its and subsequent homodimerization/activation serving as a signal to initiate mitophagy [69] (Figure 6).

#### 6.1.2. Segregation—The Link with Fission

In order for mitochondria to undergo mitophagy, targeted mitochondria must be segregated from the healthy mitochondrial network through fission. Although the molecular basis for the relationship between mitochondrial dynamics and mitophagy remain poorly understood, multiple studies have demonstrated that alterations in mitochondrial fission/fusion proteins can affect mitophagy (Figure 6). For example, inhibition of Fis, or inhibition of DRP1 via transfection with a dominant negative form of the protein, result in the accumulation of oxidized mitochondrial proteins and a decrease in mitophagy over a 10-day period [221]. This concept is supported by observations that, under conditions of mild oxidative stress induced by treatment with rotenone or H_2_O_2_, mitophagy was inhibited in cells expressing a dominant negative variant of DRP1 [199], while overexpression of Fis1 was shown to increase mitochondrial fission and, subsequently, mitophagy [222].

Recently, several studies aimed to establish whether the converse was also true; i.e., whether manipulation of proteins involved in mitophagy can alter mitochondrial morphology. In support of this concept, data obtained from primary rat hippocampal neurons demonstrated that overexpression of both PINK1 and Parkin was accompanied by mitochondrial fragmentation [223], possibly due to Parkin-mediated ubiquitination of outer mitochondrial membrane proteins involved in fission/fusion (including the mitofusins) and resultant inhibition of fusion [213,224,225]. BNIP3 and FUNDC1 have also been implicated to influence mitochondrial dynamics via complex interactions with both DRP1 and OPA1 [182,226]: i.e., FUNDC1 participates in the control of mitochondrial fusion under normal physiological conditions through its interaction with OPA1 while, in the setting of hypoxia, dephosphorylation of FUNDC1 induces dissociation from OPA1 and its subsequent association with DRP1 to promote fission [227]. Taken together, these data suggest that proteins involved in mitophagy can associate with the molecular gatekeepers of fission/fusion. It is, however, important to note: while fission is required for isolation and mitophagic clearance of defective mitochondria, mitophagy is not required to for mitochondrial fragmentation [199,213,221,228,229].

#### 6.1.3. Recognition

After a damaged mitochondrion is segregated from the mitochondrial network, the next requisite step in the execution of mitophagy is the recognition of the targeted organelle by the autophagosome. In brief, this is achieved by the conjugation of light chain 3-I (LC3-I: located in the cytosol) to phosphatidylethanolamine to form LC3-II, the recruitment of LC3-II to the phagophore (the term for the early autophagosome), and the subsequent interaction and binding of LC3-II with multiple mitophagy receptors including optineurin (OPTN), nuclear dot protein (NDP52), cardiolipin, BNIP3/NIX, and FUNDC1 [230,231,232,233]. Once activated (i.e., by mechanisms including ubiquitination [207,234,235,236,237,238]), these proteins facilitate the recognition of dysfunctional mitochondria and association with the phagophore via LC3II binding [182,217,226,239,240,241,242] (Figure 6).

#### 6.1.4. Degradation

Once the damaged mitochondria are sequestered, the autophagosome will fuse with the lysosome, resulting in the degradation of its cargo via acid hydrolase enzymes [243,244]. Autophagosome fusion is mediated by a variety of proteins including soluble NSF attachment protein receptor (SNARE) proteins, endosomal coating proteins (COPs), the endosomal sorting complex require for transport (ESCRT III) complex, the homotypic fusion and protein sorting (HOPS) complex, LAMP proteins, GTPase Rab proteins, the beclin 1 binding protein Rubicon, and chaperone HSP70 family proteins [245,246,247,248,249,250,251]. The acidic environment of the autophagosome promotes the degradation of its contents, with the resultant components (lipids and amino acids) being recycled and reused during mitochondrial biogenesis (Figure 6).

### 6.2. PINK1/Parkin and the Ubiquitin-Proteasome System

Given the E3 ligase activity of Parkin and its role in stimulating mitophagy, it has been proposed that Parkin also serves to activate the ubiquitin-proteasome system (UPS) for proteolysis of damaged OMM proteins following IR injury [214,252,253]. The UPS consists of: (1) ubiquitination of damaged or misfolded proteins by myriad of E3 ubiquitin ligases; and (2) degradation of the ubiquitinated proteins via the proteasome [254,255]. There is compelling evidence that the UPS and autophagy are activated in concert and functionally interact to maintain proteostasis in both physiologic and pathologic conditions [256,257,258,259,260]. For example, evidence obtained using quantitative proteomics demonstrated a 9-fold increase in Lys-48 (K48)-linked polyubiquitination (polyubiquitin chain recognized by the proteasome) and a 28-fold increase in K63-linked polyubiquitination (polyubiquitin chain associated with LC3 recognition) on mitochondria in HeLa cells after a 4 h treatment with CCCP—effects that were Parkin- dependent [214]. In addition, it is well-recognized that genetic and pharmacologic impairment of the UPS activates autophagy [258,261,262,263,264,265,266], in pathological conditions, such as acute myocardial MI, the extensive damage inflicted by cytotoxic ROS has been shown to generate large volume of protein aggregates that can impair proteasome activity and exacerbate myocardial damage [267,268,269,270,271,272,273,274]. Taken together, these data suggest there is a threshold for proteasome activity and, in the context of IR injury, autophagy is activated to assist in the removal of damaged proteins and organelles.

## 7. Mitophagy and Cardiomyocyte Fate

Myocardial ischemia-reperfusion has been associated with an activation or upregulation of autophagy and, more specifically, mitophagic pathways [197,275,276,277,278,279]. However, whether activation of mitophagy is a protective mechanism, or, conversely, exacerbates cell death, remains a topic of ongoing investigation.

### 7.1. Ischemia

There is a consensus that upregulation of mitophagy during ischemia confers protection [197,280,281]. This concept is, in large part, based on robust data obtained in murine models of permanent coronary artery ligation [281]. For example, Kubli and colleagues reported that, in wild-type mice, mortality post-ligation was 20% and, in animals that survived to 7 days post-MI, the hearts displayed an upregulation of mitophagy with increased expression of Parkin at the margins of the infarct [280]. In contrast, Parkin-deficient mice subjected to coronary artery ligation were characterized by an increased, 60% mortality rate post-MI and, in survivors, exacerbation of adverse LV remodeling and contractile dysfunction when compared with the wild-type cohort [280]. These results were substantiated by in vitro evidence utilizing primary adult mouse cardiomyocytes isolated from Parkin-deficient mice [280]. Parkin translocation to the mitochondria was significantly greater in response to hypoxia in wild-type versus Parkin-deficient cells [280]. Moreover, cell death following 4 h of hypoxia was exacerbated in Parkin-deficient cardiomyocytes when compared with cardiomyocytes from wild-type mice, an effect that was abrogated when Parkin expression was restored [280]. Similar outcomes were obtained with genetic deletion of two mitophagy inhibitors, p53 and TP53-induced glycolysis and apoptosis regulator (TIGAR): mitophagy was upregulated and LV contractile dysfunction was attenuated following permanent coronary artery ligation in p53^−/−^ and TIGAR^−/−^ versus wild-type mice [281]. Moreover, at 8 h post-MI, cardiomyocytes from the p53^−/−^ cohort displayed a decrease in the number of abnormal mitochondria, significant increases in LC3 puncta, together with an increase in the number of autophagosomes containing mitochondria: i.e., evidence of an upregulation in mitophagy that was attributed to ROS-induced activation of the mitophagy receptor BNIP3 [281]. Finally, for proof of concept, additional groups of p53^−/−^ and TIGAR^−/−^ mice were treated with chloroquine, an autophagy inhibitor, following permanent coronary artery ligation. Cardioprotection conferred by deletion of p53 and TIGAR was reversed, an effect that was accompanied by an accumulation of abnormal mitochondria in the ischemic cardiomyocytes [281].

### 7.2. Ischemia-Reperfusion: ‘Good Versus Evil’

Mitophagy (and, more generally, autophagy) are upregulated following relief of ischemia, with evidence that activation of these pathways may be augmented in the setting of ischemia-reperfusion when compared with ischemia alone [279]. Moreover, many (but not all) studies have concluded that upregulation mitophagy following IR is cardioprotective [182,282,283,284,285,286,287]. Seminal data in support of this concept were provided by Hamacher-Brady et al.: using a combination of isolated buffer-perfused rat hearts, isolated neonatal rat cardiomyocytes and HL-1 cardiomyocytes together with molecular manipulation of BNIP3 expression and activity, the investigators observed that: (1) BNIP3 was upregulated following IR, and, perhaps not surprisingly, (2) BNIP3 was pro-apoptotic and contributed to the IR-associated loss in mitochondrial integrity [182]. In addition, and most notably, (3) IR was accompanied by an upregulation in mitophagy that was BNIP3-dependent [182]. Although the investigators did not establish whether BNIP3 directly stimulated mitophagy or whether the upregulation in mitophagy served as a protective response against BNIP3-induced mitochondrial dysfunction, they did demonstrate that overexpression of Atg5 (a molecule that plays a critical role in autophagosome formation) significantly enhanced autophagy and reduced BNIP3-mediated cardiomyocyte death, while transfection with a dominant negative form of Atg5 had the opposite effect: i.e., exacerbated cell death [182].

Corroboration of the proposed role of increased mitophagy in protecting against lethal IR injury has been obtained by the genetic perturbation of other molecular regulators of mitophagy, including PGAM5 (positive regulator of FUNDC1) [282], CK2 (negative regulator of FUNDC1) [285], and Parkin [283,288]. For example, translocation of Parkin to mitochondria (and, more specifically, to depolarized mitochondria) was observed in HL-1 cells subjected to simulated IR. More importantly, knockdown of Parkin rendered HL-1 cells more susceptible to IR injury and exacerbated cardiomyocyte death, suggesting that Parkin plays a critical role in cardioprotection by activating mitophagy [283,288]. Knockdown of Parkin was further reported to increase infarct size in murine hearts subjected to coronary artery occlusion-reperfusion [288] and, interestingly, abrogate the infarct-sparing effect of ischemic preconditioning [283]. This latter observation extended the paradigm of ‘cardioprotection via upregulation of mitophagy’ to suggest that Parkin, and its subsequent translocation to mitochondria, purportedly contributes to the classic and well-established ability of preconditioning to protect the heart against lethal IR injury [283,286]. Finally, recent data have confirmed the concept that Parkin mediates mitophagy and is cardioprotective, but concluded that Parkin reportedly conferred protection by mechanism not directly associated with its role in mitophagy: i.e., by ubiquitination of cyclophilin D (CypD), a protein associated with the mPTP, and thereby inhibiting mPTP [289]. Collectively, these studies suggest that while Parkin (and upregulation of mitophagy) may be cardioprotective, Parkin’s role in cardioprotection following IR injury is in all likelihood multi-faceted.

Despite the overall agreement that mitophagy (and, more generally, autophagy) is activated following ischemia-reperfusion [197,279], not all studies agree that upregulation confers cardioprotection. In this regard, upregulation of autophagy (more specifically, Beclin 1-dependent autophagy) following relief of ischemia is reportedly detrimental: i.e., pharmacologic inhibition of autophagy with 3-methyladenine, and genetic knockdown of Beclin 1, significantly attenuated, rather than exacerbated, lethal IR-induced cardiomyocyte death [197,279,290,291]. The reasons for this apparent discrepancy have not been resolved, but may be explained by the observation that Beclin 1, in addition to its classic role in autophagy, also impedes autophagosomal-lysosomal fusion [292], suggesting that the improved cardiomyocyte viability seen with Beclin 1 knockdown may be a consequence of improved autophagic flux rather than inhibition of autophagy [197,292]. In addition, it has been postulated that modest and adaptive upregulation of mitophagy is protective, whereas massive upregulation of autophagy and the attendant general degradation of organelles is maladaptive and detrimental [197,279]. That is: specific, targeted and balanced activation of mitophagy may be the critical element in evoking cardioprotection [197,279,293].

In apparent contrast to this latter paradigm, and in contrast to the overall concept of ‘cardioprotection via upregulation of mitophagy’, there is evidence to suggest that suppression (rather than upregulation) of mitophagy may be protective during IR injury [294]. Using the in vivo rat model of coronary artery occlusion-reperfusion, the activity of mitochondrial aldehyde dehydrogenase 2 (ALDH2: the enzyme responsible for the metabolism of acetaldehyde and other toxic aldehydes) was modulated by pre-ischemic administration of a pharmacologic agonist and inhibitor of the enzyme, Alda-1 and Daidzin. Pretreatment with Alda-1 was, as expected, cardioprotective and reduced myocardial infarct size—an effect that was accompanied by a significant *attenuation* in the translocation of PINK1 and Parkin to the mitochondria seen in response to ischemia-reperfusion [294]. Administration of Daidzin had the anticipated, opposite effect on infarct size (i.e., exacerbated lethal IR injury) but, interestingly, did not augment PINK1 and Parkin translocation beyond that seen with IR alone [294]. These data may be interpreted to suggest that the protective effects of Alda-1 are explained by inhibiting excessive activation of mitophagy. However, Alda-1 treatment was also associated with decreased levels of ROS and partial preservation of mitochondrial membrane potential [294], raising the possibility that mitochondrial integrity was better maintained with administration of Alda-1 and thereby precluding the need for an upregulation in mitophagy. Finally, as mitophagy is a dynamic process, assessment of PINK1 and Parkin at a single, static time-point may yield misleading results: the reported attenuation of PINK1 and Parkin translocation may reflect a more efficient clearance of targeted mitochondria with Alda-1 treatment, rather than inhibition of mitophagy. To address this possibility, comprehensive temporal assessment of autophagic flux would be required [90,295,296].

## 8. Pharmacologic Targeting of Mitochondrial Morphosis to Attenuate Lethal IR Injury

The aforementioned studies are consistent with the concept that imbalances in mitochondrial quality control contribute to the pathogenesis of lethal myocardial IR injury, with the preponderance of evidence suggesting that upregulation of mitophagy in the setting of ischemia-reperfusion favors cardioprotection. If this premise is correct, then the development of pharmacologic agents capable of stimulating mitophagy may potentially yield additional new approaches (beyond those targeting mitochondrial dynamics described in Section 5) for the treatment of lethal myocardial ischemia-reperfusion injury.

There are available drugs, including rapamycin, sulfaphenazole and chloramphenicol, that are reportedly cardioprotective and have been demonstrated to upregulate autophagosome formation [284,297,298,299]. However, these agents are not selective. Indeed, at present, there are no drugs or compounds that specifically enhance mitophagy without: (1) affecting mitochondrial function, (2) inducing a potentially maladaptive upregulation of general autophagy, and (3) having other confounding, off-target molecular effects.

Multiple small molecules are in development [300], with the goal of targeting specific proteins involved in mitophagy for both investigational and possible therapeutic use. For example, an attractive strategy for the modulation of PINK1 activity is based on the identification of N^6^-furfuryl ATP (kinetin triphosphate, KTP), a neo-substrate that exhibits higher affinity for PINK1 compared with its native substrate, ATP [301]. Using HeLa cells, pretreatment with kinetin (N^6^-furfuryl adenine), the precursor to KTP, initiated an acceleration the recruitment of PINK1 to mitochondria uncoupled by subsequent application of 2-[2-(3-Chlorophenyl)hydrazinylyidene]-propanedinitrile (CCCP) [301]. Other mediators of mitophagy targeted for small molecule development include p53 (which negatively regulates Parkin and inhibits Parkin-mediated mitophagy [281,302], and mitochondrial deubiquitinases such as ubiquitin specific peptidase 30 (which specifically antagonize Parkin mediated mitophagy by removing poly-ubiquitin chains from damaged mitochondria) [303,304]. Indeed, pharmacologic stimulation of multiple pathways involved in mitochondrial clearance is being explored, including the as-yet poorly elucidated silent information regulator T1 (SIRT1) pathway [305,306,307,308,309,310], and nuclear factor E2-related factor 2 (Nrf2), which is responsible for the expression of a variety of cytoprotective genes harboring antioxidant responsive elements in their promoter region such as the mitophagy proteins p62 and NDP52 [311,312,313]. In this regard, a candidate molecule has been identified, called p62/SQSTM1-mediated mitophagy inducer (PMI), that increases expression of p62 and drives mitophagy without inducing changes in mitochondrial membrane potential [314]. PMI can reportedly induce mitophagy independently of PINK1 and Parkin and has no effect on general autophagy [314], suggesting that PMI may serves as a selective mitophagy enhancer that drives mitophagy in a pathway-independent manner. Development of PMI is, however, in the prototype stage. Moreover, none of the aforementioned molecules have, to date, been evaluated in cardiomyocyte models or in the setting of ischemia-reperfusion.

Pharmacologic inhibition of mitophagy can also be achieved with existing agents, most notably bafilomycin A1, chloroquine, and 3 methyladenine (3MA) [315]. However, despite their routine use as tools to investigate the cellular consequences of mitophagy, they suffer from the same limitation—lack of selectivity—as the currently available activators [316,317,318]. More specific peptide inhibitors are, however, in development [242].

If selective small molecule agents are successfully developed, a second caveat will apply to any future therapeutic use. A recurring theme in mitochondrial quality control is balance: excessive activation of mitophagy could result in an inappropriate decrease in mitochondrial mass and subsequent deficit in ATP production, while disproportionate inhibition of mitophagy would result in the accumulation of dysfunctional mitochondria and a shortage of substrates for mitochondrial biogenesis. Thus, in order to achieve cardioprotection, it will be critical to administer these agents in a manner that achieves targeted, balanced and controlled mitophagy.

## 9. Broad Relevance of the Paradigm: Mitochondrial Quality Control and IR Injury in Brain

The concept that mitochondrial quality control plays a causal and mechanistic role in lethal ischemia-reperfusion injury is not limited to heart. Not surprisingly, this paradigm is also a topic of interest and active investigation in other metabolically active tissues and organs that are vulnerable to IR-induced cell death—including, most notably, the brain.

Lethal IR injury in brain, caused by diverse pathologies including ischemic stroke, cardiac arrest and neonatal hypoxic/ischemic encephalopathy, is associated with severe neurological deficits and death. As in heart, mitochondrial dysfunction plays a central role in IR injury in the brain. However, as in heart, the contribution of mitochondrial quality control to the pathogenesis of IR injury in brain remains incompletely understood and, in some cases, controversial. There is a clear consensus that IR is associated with mitochondrial fragmentation in the initial hours following relief of ischemia [85,173,319,320,321,322,323], an observation that has been made following transient focal ischemia mimicking stroke [321] and following global cerebral ischemia mimicking cardiac arrest-resuscitation [85]. Increased fission has also been observed in neuronal cell culture models (including SH-SY-5Y cells, primary neurons and HT22 cells) subjected to oxygen-glucose deprivation (OGD) [173,322]. Moreover, intriguing data obtained from isolated neuronal models suggests that the temporal profile of mitochondrial fission may be complex and biphasic, with an initial, profound but transient episode of mitochondrial fragmentation during the early minutes post-reperfusion followed by a second and sustained period of fission after 2 h of reoxygenation [173]. These phenotypic data are supported by molecular evidence of increased mitochondrial expression of DRP1 and Fis1 [85,320,324,325], together with proteolysis of OPA1, a decrease in OPA1 oligomers, and decreased Mfn2 expression [85,173,320,323] following reoxygenation.

Despite these reported associations between IR, DRP1-mediated mitochondrial fragmentation and OPA1 degradation, there is no current agreement regarding cause-and-effect. In some studies, pharmacologic inhibition of DRP1 and genetic over-expression of Mfn2 are reportedly neuroprotective [322,326,327,328], while activation of DRP1 has been shown to promote fission and sensitize cells to OGD-induced neuronal cell death [329]. In contrast, in other studies, inhibition of fission with MDIVI-1 has been shown to aggravate ischemic injury [315,330].

In brain and cultured neuronal cells, as in heart and cardiomyocytes, ischemia-reperfusion (and the attendant pro-fission phenotype) is associated with a robust upregulation of mitophagy [315,331,332,333,334]. However, in contrast to the controversies in heart (see Section 7), there is overall agreement that mitophagy plays a protective role against IR injury in brain [201,315,330,331,334,335,336,337]—a concept that is corroborated by evidence that pharmacologic or genetic suppression of mitophagy exacerbates IR-induced neuronal cell death [201,315]. This issue is not, however, fully resolved: i.e., recent evidence obtained in Neuro-2a cells has revealed that activation of general macroautophagy can evoke protection, independently of mitophagy [338]. Accordingly, in brain as in heart, mitochondrial quality control is a complex and nuanced, and the rational development of neuroprotective strategies that target mitochondrial dynamics and mitophagy in the setting of lethal IR will require further molecular characterization.

## 10. Future Directions

Based on the evidence available to date, does rational mechanisms-based targeting of mitochondrial quality control represent a tenable and translationally relevant strategy to protect the heart against lethal IR injury and improve outcomes post-MI? As detailed in Section 5 and Section 8, pharmacologic inhibition of DRP1 and OMA1, as well as upregulation of autophagy, have been shown in an as-yet limited number of studies to evoke cardioprotection and reduce myocardial infarct size. While these data are potentially encouraging, it is important to recognize that our understanding of mitochondrial quality control in the setting of myocardial ischemia-reperfusion, particularly in the in vivo setting, is in its infancy. Moreover, and of greater concern with regard to the issue of therapy, the pharmacologic agents used in these studies are, in most instances, not selective (see Section 5 and Section 8).

A second caveat that warrants acknowledgement when considering the translational relevance of these data is that, among the small number of in vivo preclinical studies conducted to date, all have utilized healthy juvenile or adult animals devoid of clinically relevant comorbid conditions typically seen in patients with cardiovascular disease. The potentially confounding effect of comorbidities (including, but not limited to, aging and diabetes) on efficacy of candidate cardioprotective strategies has become a subject of discussion and concern [339,340], and may also be relevant to treatments targeting mitochondrial fission, fusion and mitophagy [341,342,343]. For example: mitochondrial NAD-dependent deacetylase sirtuin-3 (SIRT3) regulates the acetylation status of multiple mitochondrial proteins, is well-recognized to be a critical mediator of mitochondrial function (including oxidative phosphorylation) and, more recently, has emerged as a regulator of mitochondrial quality control. SIRT3 reportedly activates DRP1-mediated fission and PINK1/Parkin-mediated mitophagy via deacetylation of Forkhead box O-3 (FoxO3), and directly deacetylates OPA1 to augment the activity of the GTPase [344,345,346,347]. In addition, and as might be expected based on these data, a deficiency in the cardiac expression and activity of SIRT3 is purportedly accompanied by a fragmented cardiac mitochondrial phenotype and defects in the inner mitochondrial membranes [347]. In adult rodent hearts, SIRT3 is upregulated under conditions of cardiac stress, including myocardial IR [347,348]. However, hearts from aged (18-20 month old) mice have been reported to display: (1) a deficit in mitochondrial SIRT3 activity and protein expression when compared with juvenile and adult cohorts [349,350], an effect that was accompanied by impaired PINK1/Parkin-mediated mitophagy [350]; and, importantly, (2) an increased susceptibility to lethal IR injury [349]. Similar deficits in cardiac SIRT3, together with attendant alterations in mitochondrial morphosis and mitophagy, have also been described in murine models of type-2 diabetes [343,347] Accordingly, whether the potential infarct-sparing effect of strategies targeting mitochondrial quality control would be maintained in aging and diabetic populations (or in the setting of any relevant comorbidities) is, at present, unknown, and conclusions regarding future translational relevance would be premature.

## 11. Conclusions

Data obtained over the past ~decade have revealed compelling evidence of inextricable links between mitochondrial quality control and cell fate in cardiomyocytes (and neurons) subjected to ischemia-reperfusion. Pathologic DRP1-mediated fission and MOMP, OPA1 disruption and loss of cristae integrity, together with inefficient clearance of dysfunctional mitochondria by mitophagy, have all been implicated to play a causal, mechanistic role in lethal IR injury. Additional studies will, however, be required to: (1) identify the precise molecular event (or, in all likelihood, combination of events) that serve as the definitive ‘executioner’ in IR-induced cardiomyocyte death, and, based on these insights (2) develop novel and targeted, mechanisms-based pharmacologic therapies to improve outcomes in patients with acute MI.
